# Identifying transdiagnostic neurobiomarkers for precision major psychiatric disorders *via* machine learning: Causes, findings, and future directions

**DOI:** 10.1515/jtim-2025-0045

**Published:** 2025-12-05

**Authors:** Fay Y. Womer, Yao Xiao, Vincent Ngo, Junjie Zheng, Fei Wang

**Affiliations:** Department of Psychiatry and Behavioral Sciences, Vanderbilt University Medical Center, Nashville, TN, USA; Early Intervention Unit, Department of Psychiatry, Affiliated Nanjing Brain Hospital, Nanjing Medical University, Nanjing, Jiangsu Province, China; Functional Brain Imaging Institute of Nanjing Medical University, Nanjing, Jiangsu Province, China; Department of Psychiatry and Behavioral Neuroscience, Saint Louis University School of Medicine, St. Louis, MO, USA

**Keywords:** schizophrenia, bipolar disorder, major depressive disorder, neuroimaging, machine learning

## Abstract

The diagnostic criteria of psychiatric disorders rest solely on symptoms rather than etiologies; this led to the lack of objective biomarkers for diagnosis, differential diagnosis and treatment of major psychiatric disorders (MPDs) including schizophrenia, bipolar disorder, and major depressive disorder. Facing this bottleneck problem, we firstly started our review from the evolution of psychiatric nosology for the diagnosis of MPDs which refined from categorical to dimensional classification. Specifically, the neuroimaging was sought as an intermediate phenotype to solve the dilemma introduced from a symptom-based diagnostic classification framework. Secondly, we reviewed findings applying traditional mass-univariate methods as well as machine learning methods to identify the diagnostic and treatment neurobiomarkers for MPDs from group level to individual level, and suggested the frontal-posterior functional imbalanced pattern as a potential transdiagnostic neurobiomarker for MPDs. We further initialized the necessity of using unsupervised learning approach for MPDs subtyping prior to performing supervised learning for classification, as seeking one single neurobiological feature should be incapable of coping with the strong heterogeneity in MPDs. We concluded that the ultimate goal of MPDs subtyping is to identify an objective neurobiomarker to guide clinical practice; based on frontal-posterior functional imbalance, we developed a subtyping and precise neuromodulation strategy to achieve the establishment of a precision medicine framework. Future studies should perform more etiological investigations based on this neurobiomarker, and conduct randomized controlled trials in larger MPDs populations for further verification.

## Evolution of psychiatric nosology for diagnosing major psychiatric disorders: From category to dimensionality

Across the centuries, our conceptualization of psychiatric disorders has been shaped by our cultural, psychological, and biological understanding of mental illness. Over the past several decades, the field has markedly accelerated its pace in gaining important insight into the neurobiological processes underlying psychiatric disorders. Subsequently, the biological basis of mental illness has become more generally accepted within the scientific and lay community. This recognition has been vital for the substantial progress in diagnosis and treatment of psychiatric disorders in recent decades. Moreover, it has radically changed the outcomes for individuals with mental illness. Once relegated to asylums and institutionalized for the remainder of their lives, affected individuals can now receive treatment that significantly reduce symptoms and impairment, and carry out their lives like those without mental illness. However, despite medical advances and improved quality of life, individuals with psychiatric disorders continue to experience significant illness burden and disability.^[1]^ The major psychiatric disorders (MPDs), schizophrenia (SCZ), bipolar disorder (BD) and major depressive disorder (MDD) are leading causes of disability worldwide and contribute to substantial economic burden in direct and indirect costs including lost productivity and caregiver burden.^[2, 3, 4, 5, 6, 7, 8]^ Further treatment innovations are needed to improve the lives of individuals with psychiatric disorders such as SCZ, BD, and MDD and their families. However, conventional diagnostic criteria for psychiatric disorders appears to be limiting the progress in the treatment development. There is significant clinical heterogeneity within SCZ, BD, and MDD as they are defined now, confounding studies of MPDs and leading to inconsistent findings.^[9, 10, 11, 12]^ This slows Psychiatry’s quest to understand the neurobiological mechanisms underlying MPDs and identify potential treatment targets that will lead to improved functional outcomes in MPDs. In this review, we will discuss the diagnostic classification of MPDs and recent neuroimaging findings in MPDs that may contribute to refining psychiatric nosology and advances toward precision medicine in psychiatry.

### Establishment of a categorical classification framework

As scientific methods have advanced, psychiatric nosology has undergone major paradigm shifts towards a medical model with empirically defined criteria and a unified language in efforts to develop a reliable and valid diagnostic framework. Psychiatric taxonomy continues to evolve as the field reconciles scientific findings with the various aspects of psychopathology, including cultural and environmental factors, and refines its diagnostic classification to enhance both clinical care and research.^[13,14]^ Diagnostic classification is of the utmost importance in any medical field. As stated in a 1988 report by Andreasen *et al*., “The ultimate goal of any system for diagnosis or clinical description is to provide insights into the etiology and pathophysiology of a given disorder. With a clear understanding of etiology and pathophysiology, effective therapy and ultimately prevention can take place.”^[15]^

The diagnostic and statistical manual of mental disorders (DSM) and international classification of diseases (ICD) are two major classification systems for psychiatric disorders used by clinicians and researchers worldwide, with the former predominantly used in psychiatry. The DSM has had multiple revisions since its first edition, DSM-I, in 1952. Released in the 1980s, DSM-III and DSM-IV revolutionized psychiatric diagnosis and propelled the field forward as a medical specialty and in empirical research.^[14]^ Both DSM-III and DSM-IV approached psychiatric diagnosis in a categorical framework and established empirically based criteria that significantly improved diagnostic reliabilityand validity.^[13,14,16, 17, 18, 19]^

### Exploration of a dimensional classification framework

Notably, the latest revision of DSM, has moved towards a more dimensional approach to psychiatric diagnosis but still largely retaining a categorical framework. For example, DSM-5 combined the DSM-IV categories of alcohol dependence and abuse into a single disorder (alcohol use disorder) measured on a continuum of mild, moderate, and severe based on symptom counts.^[20]^ DSM-5 aimed for clinical utility and made significant changes to DSM-IV diagnostic categories and criteria.^[21]^ Following its release in 2013, DSM-5 sparked much debate and controversy in how the field approaches and defines psychiatric disorders and what is considered acceptable diagnostic reliability for clinical and research use.^[13,14,20,22,23]^ DSM-5 field test was expected to yield lower diagnostic reliability than DSM-IV as it focused on clinical utility and used different methods than prior DSM field tests.^[24]^ Results from the DSM-5 field tests found significant variability in reliability across for diagnoses such as bipolar I disorder and schizophrenia. Moreover, reliability was within questionable and unacceptable ranges for two common psychiatric disorders, major depressive disorder and generalized anxiety disorder. The contribution of DSM-5 to inducing psychiatric taxonomy remains unclear. At the time of preparing this review, the release of the DSM-5, text revision (DSM-5-TR) was anticipated in March 2022. Extensive clarifications and updates to diagnostic criteria and nomenclature based on scientific literature were expected, as well as the addition of a new diagnostic category (prolonged grief disorder). The implications of DSM-5-TR for clinical care and research remains to be seen. However, while etiologic understanding of psychiatric disorders has grown over recent decades with scientific and technological advances, such as magnetic resonance imaging (MRI) and machine learning (ML), Psychiatry still lacks diagnostic biomarkers or quantitative measures. Thus, DSM nosology remains based solely on clinical phenomenology and reliant on subjective reports and clinician observation, which are prone to causing errors, misperception and misattribution.^[25,26]^

### Dilemma over diagnosing major psychiatric disorders

Diagnostic classification is particularly challenging in psychiatry as emotions and behaviors occur along a continuum and “zones of rarity” are uncommon.^[27]^ Moreover, what emotional state or behavior are deemed “pathological” depends on the context in which they occur and require understanding of typical responses or behaviors within a given culture or society. Without diagnostic biomarkers and quantitative measures, setting thresholds for disorders (*i.e*., demarcating disease from healthy) can be arbitrary and subjective in psychiatry. Consequently, in the present day, psychopathology is often recognized and definitively diagnosed when it becomes severe (*e.g*., the presence of delusions and hallucinations, disorganized thoughts, and suicidal behaviors) or after a protracted period of observation. This, in turn, results in delayed treatment and intervention and increased disease burden on affected individuals and their families, as well as poorer outcomes and potential disease progression in the brain.^[28, 29, 30]^ Inclusion of biomarkers and quantitative measures in psychiatric nosology is much needed. Such inclusion would reduce the field’s reliance on subjective reports and observations for diagnosis and facilitate more precise classification of psychiatric disorders.

SCZ, BD, and MDD have long been considered separate diagnostic entities. Their conceptual roots are founded in Kraepelinian dichotomy, which was introduced in the late 1800s.^[31,32]^ Emil Kraepelin proposed two major divisions of psychoses: dementia praecox and manic depression. Dementia praecox became known as SCZ, and manic depression as BD, as defined in DSM-III and DSM-IV. Thus, Kraepelinian dichotomy has persisted to modern day as a core basis for diagnostic classification of MPDs. However, questions have lingered about the validity of Kraepelinian dichotomy and whether it should continue to shape how MPDs are conceptualized^.[31, 32, 33]^ Decades of research have yet to confirm SCZ, BD, and MDD as separable diagnostic entities based on genetic, molecular, neurobiological, and clinical studies. Conceivably, DSM constructs are not sufficiently homogeneous to reduce noise-to-signal ratio for studies to detect distinct differences across SCZ, BD, and MDD. Alternatively, DSM classification may not align well with biological distinctions among MPDs.

### Seeking neuroimaging as an intermediate phenotype

Fortunately, further progresses have been made by DSM-5 through the combined diagnoses of category and dimensionality. The dimensional approach situates psychiatric disorders in a spectrum with partial overlaps across diseases and shared underlying causes within dimensionalities.^[34]^ Although the implication of dimensionality remains premature in clinical practice, it offers us an opportunity to rethink the MPDs pathogeneses through a hierarchical continuum. In 2010, Craddock *et al*. proposed a dimensional spectrum in which five clinical syndromes including the MPDs lied on a single axis;^[32]^ further, as MPDs arose from a diverse array of genetic, environmental factors and their interactions, each dimension reflected distinct genotypes and clinical phenotypes.^[35]^ However, it’s not the behavior domain but the more fundamental cellular processing that the gene directly encodes; together, the genome, transcriptome, proteome, metabolome, neuroimaging, and symptomatology compose of a hierarchy across the MPDs casual pathway.^[36]^

**Figure 1:**
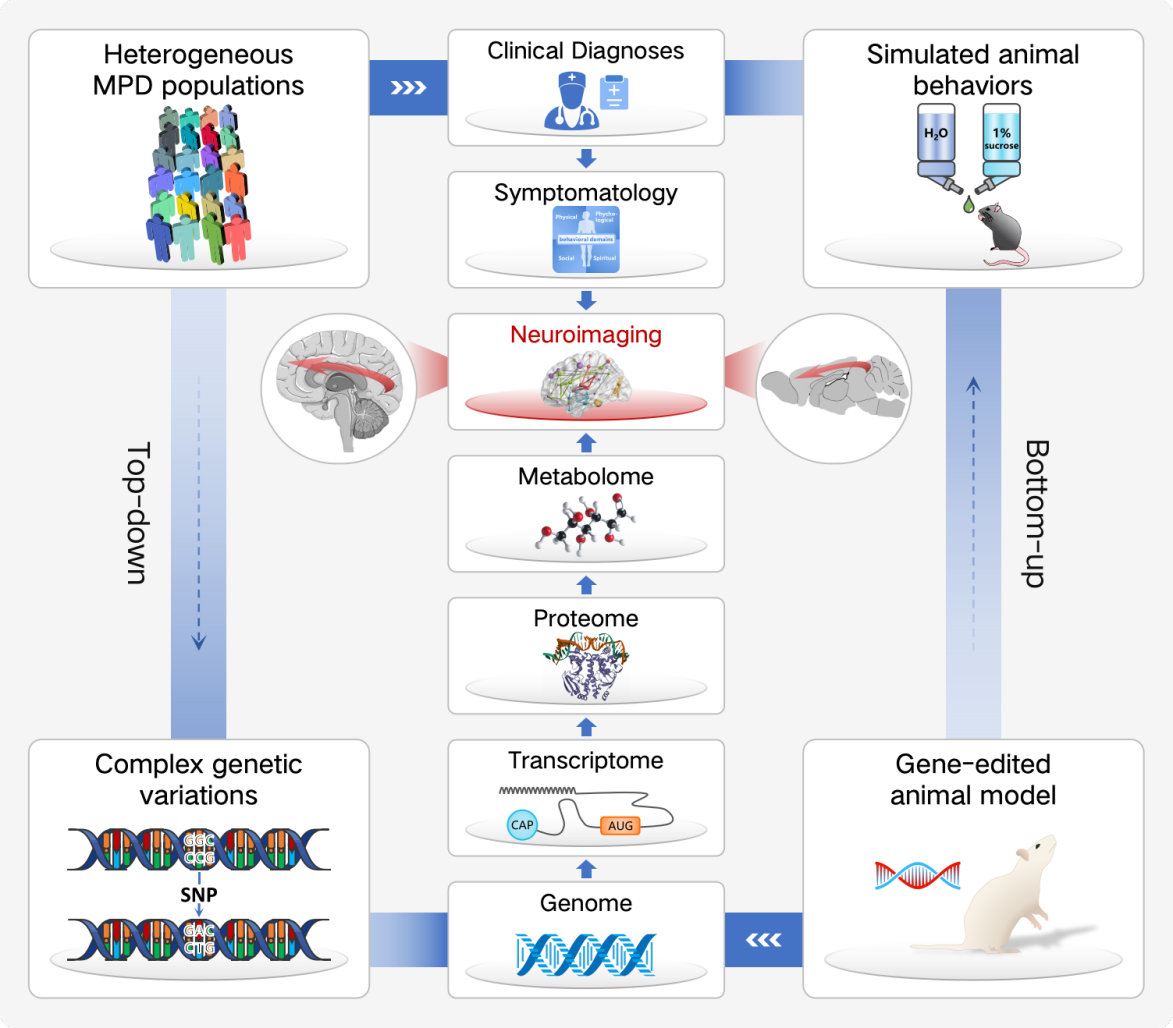
Neuroimaging is increasingly being identified as a cross-species intermediate phenotype, linking together the multi-level casual pathway of major psychiatric disorders (MPDs) for a better translation from animal model to clinical implications.

Pathogeneses of MPDs remain to be explored. As a consequence, one well-established strategy for psychiatrists to understand the biological underpinnings of MPDs could be characterized as bottom-up (from genes to symptoms), in which case the animal model serves as an important tool.^[37]^ For example, the P11 knockout model is one of the most frequently used animal model for MDD simulation, whose employment of gene-editing technique enables us to directly investigate the MDD etiologies in a casual way across the longitudinal life span. Nevertheless, the behavioral similarity between animal models and human patients remain non-confirmed. On the other hand, the top-down, symptom-based approach proceeds straight from the MPDs population; however, the diagnoses which evolved from the clinical needs rather than biological validity make it insusceptible to investigate the complex genetic variations regarding the heterogeneous MPDs.^[37]^ Considering the multi-level pathogeneses of the MPDs, an intermediate phenotype which serves as a linkage and be capable of transiting lower to genes as well as higher to symptoms would be of great necessity.^[38]^ Since the psychiatric disorders are increasingly reconceptualized as brain circuit disorders, the neuroimaging has become a potential transdiagnostic biomarker that modulates different cognitive and emotional domains, as demonstrated by the Research Domain Criteria project (RDoC).^[39]^ Taken together, the neuroimaging is identified as a cross-species intermediate phenotype to refine MPDs in an objective and etiological way, linking together the multi-level casual pathway for a better translation from animal model to clinical implications (Figure 1).

Specifically, mounting evidence has shown significant similarities and overlaps across MPDs in genetic risk, molecular and neural alterations, symptomatology, cognition, and behavior, with a general trend of greatest severity in SCZ, followed by BD and then MDD (SZ > BD > MDD).^[40, 41, 42, 43]^ Moreover, previously hypothesized to stem from a single or small subset of neural substrates, MPDs have now been shown to involve a multitude of brain regions and networks spanning across the whole brain.^[44, 45, 46, 47]^ As the complexity of brain networks and MPDs has unfold, it is increasingly evident that studies of MPDs should look beyond group-level differences and examine individual variability in brain structure and functional brain networks as well.^[48, 49, 50, 51, 52]^ Group-level comparisons essentially create an “average” for each group, and resultant findings are dependent on how the groups were defined. Consequently, these types of comparisons could mask or conflate critical details with implications for understanding the neural mechanisms underlying MPDs, investigating pharmacological treatment effectiveness, identifying novel treatment targets, and predicting treatment response. ^[50,53]^ Altogether, prior findings highlight the diagnostic and neurobiological complexity of MPDs and indicate a greater significance of dimensional features among MPDs than previously considered. Neuroimaging acts as an ideal intermediate phenotype to delineate the neurobiological complexity and dimensionality of MPDs and can be meaningfully incorporated in psychiatric nosology.^[14, 15, 16, 17, 18, 19, 20, 21, 22, 23, 24, 25, 26, 27, 28, 29, 30, 31, 32]^

## Developmental history of applying neuroimaging in major psychiatric disorders: From group to individual

Advances in neuroimaging techniques, particularly MRI, have opened a window into the brain’s structure and inner workings in a noninvasive and safe manner. MRI uses powerful magnets to capture images of the brain in three dimensions (axial, coronal, and sagittal). Different scanning parameters are used to detect various properties of the brain, such as gray and white matter differentiation, water diffusion along axonal white matter, and changes in blood oxygen. Data collected during scanning is then processed and computed to create images for use in clinical and research settings. Below is an overview of different MRI techniques and measures:

- Structural MRI (sMRI): Provides detailed images of brain structure showing gray and white matter and the intricate folding of brain structures.
  - *Voxel-based morphometry (VBM)* identifies volume differences between groups using spatially normalized images.^[54]^
- Diffusion tensor imaging (DTI): Uses water diffusion to provide measures reflecting white matter microstructure.
  - *Fractional anisotropy (FA)* measures the degree of anisotropy of water and is thought to reflect fiber density, axonal diameter, and myelination of white matter.^[55]^ Higher FA indicates movement of water along a single axis (*i.e*., axonal fiber).
  - *Tract-based spatial statistics (TBSS)* is a method for analyzing and interpreting diffusion results using non-linear registration and projection onto an alignment-invariant tract representation (*i.e*., “mean FA skeleton”).^[56]^
- Resting-state functional MRI (rs-fMRI): Uses blood oxygen level dependent (BOLD) signals to provide measures reflecting brain function and their synchronization while the subject is resting and not engaged in a task.
  - *Amplitude of low frequency (0.01-0.08 Hz) fluctuations (ALFF)* indicates the regional intensity of spontaneous fluctuations in BOLD signals and is thought to reflect spontaneous neuronal activity within a given region.^[57,58]^
  - *Functional connectivity (FC)* indicates the temporal correlation of BOLD signals between spatially distant points.^[58, 59, 60]^
  - *Regional homogeneity (ReHo)* indicates the synchronization of time series for BOLD signals of a given voxel and its nearest neighbors.^[58, 59, 60]^

As with any research technique, there are caveats and limitations to MRI. Methodological differences in data acquisition and processing (*e.g*., scan parameters and duration, motion correction, and noise reduction) may skew results and contribute to inconsistent findings between studies, as well as hinder comparisons between studies.^[61]^ MRI contraindications (*e.g*., having a metallic implant or pacemaker) and scanning conditions (*e.g*., individuals must lay still in a tight and enclosed space for an extended duration) may lead to selection bias (*e.g*., exclusion of certain psychiatric conditions, clinical severity, or age groups associated with poor tolerability of MRI).

### Application of traditional mass-univariate methods in neuroimaging analyses

#### Diagnoses and differential diagnoses of major psychiatric disorders

Over recent decades, a substantial wealth of MRI data has accumulated in SCZ, BD, and MDD. Despite numerous MRI studies in MPDs, including comparison and transdiagnostic studies, neurobiological markers that clearly differentiate SCZ, BD, and MDD remain elusive. Studies comparing SCZ and BD, and BD and MDD are more common, while those comparing SCZ and MDD are limited. Clinical heterogeneity within diagnostic categories and confounding factors such as medication exposure, illness chronicity, and age have complicated interpretation and generalizability of findings in MPDs and likely contribute to inconsistencies among studies^.[62, 63, 64]^ These challenges underscore the need for further parsing of the heterogeneity within MPDs, in addition to studies of affected individuals during early stages of illness with no or minimal exposure to psychiatric medications. However, it remains unclear which disease features, such as genetic or neuroimaging markers, may reduce MPDs heterogeneity in a clinically meaningful way.

Widespread structural alterations have been shown in SCZ and BD, including in the prefrontal cortex (PFC), temporal lobe, parietal lobe, cingulum, amygdala, hippocampus, thalamus, basal ganglia, and cerebellum, based on similar findings of both our and other research group.^[41,64, 65, 66, 67, 68, 69]^ Further, one of our prior studies investigated the structural changes between first psychotic episode SCZ. Their first-degree relatives (*i.e*., genetic high risk, GHR) and healthy controls (HC) as SCZ is a highly heritable disease. Compared with HC, both SCZ and GHR revealed significantly increased GMV in the right middle frontal gyrus and inferior operculum frontal gyrus, indicating which to be potential neurobiomarkers for the vulnerability of SCZ at an early stage.^[70]^ Meanwhile, structural alterations in MDD appear less extensive than in SCZ and BD and have been shown in the PFC including dorsolateral, medial, and ventrolateral PFC and orbitofrontal cortex (OFC), anterior cingulate, hippocampus, and amygdala.^[71]^ With respect to direct comparisons, there are more studies comparing SCZ and BD, and BD and MDD than SCZ and MDD.^[65]^ In SCZ versus BD, structural alterations appear more extensive in SCZ than BD, and longitudinal studies have generally found greater reduction in brain volumes over time in SCZ than BD.^[65,72]^ However, structural features that more specifically differentiate SCZ and BD have not been identified, with some structural studies finding minimal or no differences when directly comparing SCZ and BD.^[41,72]^ Comparison studies of BD and MDD have largely shown no differences in brain volumes, although volume differences have been shown in the cingulate gyrus and hippocampus.^[65]^ Prior studies of SCZ versus MDD have shown differential volume decreases in the PFC and hippocampus, with some inconsistencies in hippocampal findings.^[65]^ However, structural studies comparing SCZ and MDD are quite limited and generally have been of small sample sizes. Therefore, definitive conclusions regarding structural distinctions between SCZ and MDD cannot be drawn.

DTI studies have shown FA alterations in several major white matter tracts, including the corpus callosum, cingulum, superior longitudinal fasciculus, inferior frontooccipital fasciculus, uncinate fasciculus, thalamic radiations, and internal capsule, in SCZ, BD, and MDD, consistent with other findings of white matter abnormalities in MPDs.^[73, 74, 75]^ These findings indicate the significance of structural dysconnectivity in MPDs and support the conceptualization of MPDs as brain network or connectome disorders.^[45,76,77]^ In comparing SCZ, BD, and MDD, more similarities than differences in FA alterations have been shown across the three diagnostic categories by both our studies and other research groups, especially between SCZ and BD.^[65,78,71]^ One of our prior study of the corpus callosum found shared FA alterations between SCZ and BD in anterior, middle, and posterior genu, as well as the posterior body and the anterior splenium.^[79]^ Further, a recent meta-analysis found that the extent of FA alterations appeared greater in SCZ compared to BD and MDD.^[80]^ In a study of gray and white matter structure across SCZ, BD, and MDD using VBM and DTI, we found significant differences in gray matter volumes in 12 clusters across SCZ, BD, MDD, and HC.^[41]^ These clusters included OFC, dorsolateral PFC, cuneus, paralimbic regions such as temporal pole, insula, and pre-central, inferior parietal, inferior occipital, cingulate, and parahippocampal gyri. Of these 12 clusters, SCZ, BD, and MDD shared significant decreases in gray matter volume, compared to HC, in six clusters comprising 87.9% of the total volume with significant differences in the four-group analyses. The six clusters consisted of OFC, dorsolateral PFC, temporal pole, cuneus, insula, and cingulate, parahippocampal, and angular gyri. Post-hoc analyses of significant differences in gray matter volume across SCZ, BD, and MDD revealed shared decreases between SCZ and BD, SCZ and MDD, and BD and MDD, when compared to HC. They also found significant differences in gray matter volume when compared to HC only in BD. Altogether, these VBM findings indicate substantial gray matter alterations shared across SCZ, BD, and MDD. DTI analyses by us showed significant FA differences in five clusters across SCZ, BD, MDD, and HC. These clusters consisted of corticocortical, limbic-paralimbic-heteromodal, thalamo-cortical, callosal, and cerebellar white matter, corresponding to the cingulum, corpus callosum, fornix, superior longitudinal fasciculus, uncinate fasciculus, posterior thalamic radiation, internal capsule, and cerebellar peduncles. Post-hoc analyses showed significantly decreased FA in all five clusters common to SCZ and BD, when compared to HC. No significant differences in FA were observed between MDD and HC in any of the five clusters. These findings implicate greater involvement of white matter alterations in SCZ and BD than MDD, as well as the role of several major white matter tracts in SCZ and BD.

Functional studies provide further perspective of the neural mechanisms underlying MPDs, including brain function at rest and during specific tasks, as well as regional and distal characteristics of brain function. Initial fMRI studies examined activation within specific brain regions at rest and during tasks in SCZ, BD, and MDD. Task-based fMRI studies in MPDs have involved face emotion and reward processing, and cognitive tasks, such as working memory and executive function. In recent years, advances in fMRI techniques have allowed study of the temporal correlation or synchronization of spontaneous neural activity between and within brain regions at rest in MPDs. Notably, resting-state fMRI studies offer important insight into task-independent disease traits and the functional architecture of the brain, elucidating the brain’s functional interconnections and major hubs of neural activity at rest.^[58,82]^ As fMRI techniques have developed, there have been technical challenges (*e.g*., adequate scanning duration and data preprocessing to reduce noise and confounding effects) and debate as to how results should be interpreted (*e.g*., the physiologic and clinical implications of specific neuroimaging measures).^[58]^ Consequently, reproducibility of findings and clinical validity of measures are priority concerns.

Functional alterations appear to be widespread in SCZ, BD, and MDD at rest and during tasks, involving regions such as the PFC, parietal and temporal lobes, precuneus, anterior cingulate, amygdala, hippocampus, and thalamus, as has been repeatedly found in our and other groups’ studies.^[81,83, 84, 85, 86, 87, 88]^ Task-based fMRI comparisons of SCZ, BD, and MDD are limited but have shown similarities and differences between the three diagnostic groups.^[65,89,90]^ Similarities and differences have also been observed across SCZ, BD, and MDD in resting-state fMRI studies. Significant alterations in frontoparietal, fronto-amygdalar, corticothalamic, and cerebello-thalamic FC have been shown in SCZ, BD, and MDD, consistent with structural connectivity findings in MPDs as previously discussed.^[65,81,91, 92, 93, 94, 95, 96]^ Similarities and differences in FC of the amygdala and anterior insular cortex have been shown when comparing SCZ and BD, and SCZ and MDD.^[42,91, 92, 93, 94, 95, 96, 97]^ These shared and distinct FC alterations support the presence of core neurobiological features underlying SCZ, BD, and MDD and the PFC as a key region for differentiating SCZ, BD, and MDD, consistent with findings from different lines of study.^[95, 96, 97, 98, 99, 100, 101, 102, 103]^ Similarities and differences across SCZ, BD, and MDD have also been observed in distance-based FC analyses.^[43]^ Our group previously found similarities in alterations involving short- and medium/long-range connections across SZ, BD, and MDD. Significant alterations in short-range connections were observed in primary sensory and association cortices and the thalamus, while those in medium/long-range connections primarily involved in the PFC. Further the extent of alterations appeared to be in gradient manner across SCZ, BD, and MDD: SCZ > BD > MDD. Alterations in medium/long-range connections also had differential localization within the PFC across SCZ, BD, and MDD with greater similarities between SCZ and BD. SCZ had altered medium/long-range connections in bilateral dorsolateral PFC and left OFC, while BD had alterations in bilateral dorsolateral PFC and MDD had alterations in right dorsolateral PFC. In addition, SCZ and BD had alterations in medium/long-range connections in left supplementary motor area. Furthermore, we also investigated the distance-based dysfunctions of SCZ patients and their GHR. Compared with HC, SCZ and their GHR similarly showed significantly lower short-range connections in right lingual and left postcentral gyrus, while oppositely showed higher & lower medium/long-range connections in left OFC.^[104]^ These findings suggested the primary and higher-order cortices to be the vulnerable and resilient factors of MPDs during disease development. Taken together, the distance-based FC findings indicate the importance of both regional and distal characteristics of brain function in MPDs.

Recent studies have examined regional characteristics of brain function in MPDs using resting state measures, and ReHo. Similar to FC studies, we have also found significant ALFF and ReHo alterations in regional brain function in SCZ, BD, and MDD, as well as shared and distinct features across the three diagnostic groups.^[105, 106, 107, 108, 109, 110]^ Affected regions are similar to those altered in structural and FC studies, including the PFC, hippocampus, striatum, thalamus, and primary sensory and motor cortices such as the visual cortex. Importantly, ALFF and ReHo alterations have been associated with symptomatology and cognitive impairments in SCZ, BD, and MDD, supporting the validity of our findings.^[109,111, 112, 113, 114]^ In addition, we have found ALFF alterations in inferior frontal gyrus to positively correlate with polygenic risk scores for SCZ (PRS-SCZ) in SCZ.^[115]^ Genes associated with PRS-SCZ are highly enriched for those involved in synaptic organization and transmission, particularly for glutamatergic synapses. Moreover, our ALFF and ReHo studies have shown functional imbalance between frontal and posterior brain in MPDs.^[105,107,111]^ Besides, cross-species study indicated the imbalanced pattern to own a longitudinal neurodevelopmental process, with the posterior brain regions showing ReHo alterations during adolescence, followed by a progressive frontal changes in adulthood.^[114]^ Excitingly, our recent transdiagnostic study of SCZ, BD, and MDD has further implicated possible MPDs subtypes based on frontal versus posterior ALFF patterns, highlighting the potential importance of neuroimaging patterns in advancing our understanding of MPDs.^[105]^ Further studies are needed to elucidate the clinical relevance of the frontal versus posterior functional imbalance.

#### Pharmacological treatments for major psychiatric disorders

In addition to diagnosis and differential diagnosis, neuroimaging studies have also been implemented in guiding pharmacological treatment for MPDs. The current treatment strategies for MPDs are as follows: the selective serotonin reuptake inhibitors (SSRIs) are recognized as the first-line antidepressant medications for MDD, while the only evidence-based class of antipsychotic drug works by blocking the D2 postsynaptic dopamine receptors.^[116, 117, 118]^ Meanwhile, treatment of BD is more complicated and the first step is to define the mood state since the usage of medications differ considerably across episodes. For acute management, bipolar depression would be more challenging than mania, and mood stabilizers, antipsychotics as well as their combinations are generally adopted as the mainstay; on the other hand, the lithium has been highlighted as one of the most effective long-term maintenance treatment.^[119]^ However, the precise mechanisms of pharmacological treatment remain uncertain, and the selection of medications is limited to empiricisms, thus making the treatment effectiveness of MPDs suboptimal. Using neuroimaging in pharmacological studies could help psychiatrists investigate brain structural and functional changes post medication, predict treatment responses or refractories, and even explore the potential neural mechanisms of MPDs. Nevertheless, confounding factors such as medication classes, durations, histories, responses, and differences in control groups (healthy control, medication-free patients, placebo control, *etc*.) make existing comparative studies mixed in results. Further, the neuroimaging study itself also obtains limitations such as small sample size and varied scanning parameter. Hence, the clinical implications of current neuroimaging studies require further explorations.

Despite all these potential issues, relatively consistent conclusions could still be drawn. Compared with fMRI or DTI, the sMRI seems to be more susceptible to medications. Antidepressants and mood stabilizers, especially for lithium, have similar changes on brain structures; post medication, the structural alterations of frontal-limbic regions (including the amygdala, hippocampus, dorsal lateral and medial PFC, OFC and anterior cingulate cortex) would be normalized, corresponding to predictions of pharmacological responsiveness.^[120,121]^ Effectiveness of antipsychotics on structural alterations, on the other side, is much more complex. As demonstrated earlier, BD and SCZ shares common underlying neural mechanisms, and atypical antipsychotics are widely used for the acute as well as long-term managements on the psychotic symptoms. Evidences suggest that short-term atypical antipsychotics maintain or even increase cortical thickness, while longterm treatments lead to structural loss. The later results, however, are lack of reproducibility, possibly because of the difficulty in conducting clinical trials for over a year.^[122]^ We therefore conducted two real-world studies to investigate the effects of long-term (mean durations both longer than 12 months) atypical antipsychotics on cortical thickness and brain volumes in BD and SCZ using unmedicated patients and HCs as comparisons.^[107,123]^ For patients who received long-term atypical antipsychotics, similar to prior findings, significant cortical thinning and gray matter volume loss were widely found in the frontal and temporal lobes, cingulate gyrus and insula; further, as few study has mentioned about, these alterations are age-related. Both human and animal studies suggest that the cumulative effects of long-term atypical antipsychotics could cause reductions in glial cells, neurites and synapses, which could be the potential mechanism of structural loss in medicated patients.^[124,125]^ Moreover, the brain structure loss is positively correlated to both symptomatic improvements and cognitive impairments, indicating that atypical antipsychotics potentially contribute to a complex, adaptive as well as maladaptive compensatory effectiveness.

#### Findings and challenges

Prior structural and functional MRI findings implicate wide involvement of various brain regions including the PFC, limbic, thalamus, and primary sensory and motor cortices and several major white matter tracts such as the corpus callosum, cingulum, superior longitudinal fasciculus, and inferior fronto-occipital fasciculus in SCZ, BD, and MDD. Structural and functional connectivity studies also underscore the importance of regional and distal brain connectivity across SCZ, BD, and MDD, supporting the concept of SCZ, BD, and MDD as brain connectome or network disorders. Further, patterns of brain alterations may have important implications in identifying subtypes in MPDs and parsing the heterogeneity within diagnostic groups. Besides, the structural MRI acts as a state (rather than trait) neurobiomarker of MPDs and is significantly influenced by pharmacological treatments, with the types and durations of medications playing a vital role amongst. The structural alterations after long-term medications might not be the causes of symptomatic improvements, but compensatory effects of brain deficits.^[107,123]^ Altogether, prior MRI findings further emphasize the complexity of neural mechanisms underlying MPDs psychopathology and the challenges in incorporating neurobiological features in clinically meaningful ways. Recent advances in ML may be critically useful in analyzing the wealth and complexity of data in MPDs to improve our understanding of these disorders and advance treatment development in psychiatry, including precision or personalized medicine.

### Application of machine learning methods in neuroimaging analyses

Why should we use ML? Over decades, traditional mass-univariate neuroimaging approaches have revealed brain functional and structural abnormalities in MPDs. Although these findings help us to learn about general or regional brain deficits of MPDs, it has not been possible to use these group-level inferences to make diagnostic and treatment decisions about individual patients.^[113,114]^ ML offers a set of tools that promises to overcome this issue by mining structured knowledge or patterns from the imaging data, are particularly suited to achieve individual-level clinical identifications and predictions.^[126]^ Its members include supervised methods, such as support vector machines and neural-network algorithms; and unsupervised methods, such as algorithms for data clustering and dimensionality reduction. Notably, despite that various attempts have been made in using ML to guide the diagnosis, prognosis and treatment of MPDs, the clinical translation remains limited and several obstacles are yet to been passed. Due to the very nature of having/ lacking class labels, the supervised methods are mostly applied for classification in MPDs while the unsupervised methods provide a suitable pathway for disease subtyping. In this review, we mainly focus on these two prospects by discussing current findings and their potential limitations.

#### Supervised learning methods for classifying major psychiatric disorders

In this part, we discuss important considerations when evaluating the application of ML to MPDs, particularly focusing on supervised learning approaches. Supervised ML identifies relationships between multivariate features (*e.g*., functional connections, gray matter volumes) and subject labels (*e.g*., patient *vs*. control or subtype groups) using a learning algorithm (*e.g*., support vector machines). There are a number of supervised learning algorithms (*e.g*., k-nearest neighbor, support vector machines, decision trees) that combine information across features in different ways. Algorithm selection depends on many factors including the research question, type of data, and nature of the training data. In general, ML procedures involve training (*i.e*., feature selection, feature weight optimization, and cross-validation) and testing (*i.e*., model performance, model generalizability). The patterns of features that best classify individuals in the training set according to their labels are weighted and combined in a resulting classifier that can be applied to a distinct set of individuals in the testing phase. While most studies trained the classifier which often implies a binary model (e.g. patient or control), multivariate categorical model (*e.g*., disease 1, disease 2, … and control) was more valuable for using it into clinical practices.

Evaluation of the model is typically assessed by testing how well a classifier can predict the labels of a set of individuals never used for training, either across folds of cross-validation or in an independent validation set. Cross-validation (CV) addresses this by repeating the train/ test steps on different splits of the data. There are several forms of CV, each defined by the method for generating these splits; we will focus on the most common, generally best performing, form: *k*-fold CV. In *k*-fold CV, the dataset is evenly split into k test-sets. The prediction model tested on such a left-out set is trained on the remaining *k*−1 sets combined. The average performance gives an estimate of the quality of models produced with this training algorithm. A special form of k-fold CV is when *k* = *N*, yielding N test sets of size 1; this procedure is called leave-one-out (LOO) CV. When considering clinical use of ML models, whether the models can maintain these accuracies when being applied to brain images acquired using different scanners and in different populations should be tested. While most studies used CV in model evaluation, only a few studies used independent test samples to perform a multi-center study.^[127,128]^ The advantage of these approaches is that they better test the generalizability of the prediction models.

Several ML studies have attempted to use brain functional and anatomical data to distinguish patients with MPDs from healthy individuals, with promising results.^[129]^ A previous comprehensive literature review that search for research articles performing MRI-based predictive analyses of psychiatric illnesses showed that more than 100 ML studies in SCZ and followed by MDD and BD. The Most of ML studies in SCZ were base sMRI data than other rsfMRI, task-fMRI and dMRI data.^[129]^

**Schizophrenia research**: Based on specific changes in brain volume, several groups have shown that ML can distinguish non-medicated, first episode patients with schizophrenia from healthy controls using volumetric MRI data.^[130]^ Although previous findings could suggest 80%+ classification of SCZ and HC, only a few of studies have the independent data to validate the stability of the classification model.^[130,131]^ In recent multicenter analysis, they tested both traditional ML and an emerging approach known as deep learning (DL) using 3 feature sets of interest: surface-based regional volumes and cortical thickness, voxel-based gray matter volume (GMV) and voxel based cortical thickness (VBCT). To assess the reliability of the findings, we repeated all analyses in 5 independent datasets, totaling 956 participants (514 FEP and 444 within-site matched controls). The performance was assessed *via* nested CV and cross-site CV. Accuracies ranged from 50% to 70% for surfaced-based features; from 50% to 63% for GMV; and from 51% to 68% for VBCT. The best accuracies (70%) were achieved when DL was applied to surface-based features.^[132]^ In functional MRI studies, One of the first relatively large-scale study (*i.e*., sample size > 150) classified SZ using three different task-based fMRI data (*i.e*., auditory oddball, Sternberg item recognition and sensorimotor tasks) of 155 participants from two sites.^[133]^ In resting state fMRI study, Overall, although the sample size was relatively large across these studies, with classification accuracies ranging from 62% to 100%, the results might not be generalizable across other studies. The dMRI classification studies that utilized fractional anisotropy (FA) maps and structural connectivity from ROI as features, reported accuracies ranging from 62% to 96%, with classifiers such as SVM, LDA, Fisher’s LDC or combination of multiple classifiers^.[134, 135, 136, 137]^

In multimode studies, recently, by using measures from multimodal MRI data, Sui *et al*. extracted GMDs from sMRI, FA from dMRI and ALFF from rsfMRI to classify SCZ and HC,^[138,139]^ Lee *et al*. using structural measures (volume and/or FA) classify SCZ and HC with a 87% accuracy.^[140]^ Liu *et al*. also proposed multi kernel SVM method to classify SCZ and HC with a 90% accuracy.^[141]^ These multimode studies had a relative higher accuracy by using more informative brain features, but the sample sizes of these studies are rarely small and the framework might have introduced classification bias with lack of generalizability with independent study sample. Another multicenter analysis, Li *et al*. used functional MRI to calculated striatum functional ALFF and FC to classified patients with schizophrenia and health controls, and they had 80%+ accuracy.^[128]^ In general, we suggest the multimode features and multicenter validation data set should be involved in the future ML studies.

**Mood disorder MDD/BD research**: Some studies utilized gray matter maps as features to classify MDD and HC, which the sample size were very small.^[142,143]^ Only a few of studies had the independent validation data.^[144,145]^ Task-based fMRI ML studies were performed in previous papers. Marquand *et al*. predicted MDD with 67.5% accuracy in verbal memory N-back task.^[146]^ Fu *et al*. got 84% accuracy in sad facial processing task.^[147]^ And Johnston, *et al*. got a more accurate result in instrumental loss-avoidance and win-gain reinforcement learning task, 97% accuracy.^[148]^ Shimizu *et al*. got 95% accuracy in semantic and phonological verbal fluency task.^[149]^ Among the rsfMRI studies, region-based features from rsfMRI were used for classification MDD and HC, which can achieved 95% accuracy.^[150]^ Further, using voxel features (*e.g*., ReHo, FC) from rsfMRI data, these classification could achieve up to 90% accuracy.^[151,152]^ Diffusion MRI: Studies utilizing features from dMRI data include SVM classifier based pipelines and features such as anatomical connectivity, graph matric of white matter connectivity and voxel features (*e.g*., FA), with classification accuracies ranging from 63% to 91%.^[153,155]^ In multimodal studies, Ota *et al*. used sMRI and dMRI data and LDC classifier and obtained about 80% accuracy, although this study only work in female population, they distinguished MDD from SZ and HC individuals.^[156]^ Another large-scale MDD study (total sample size = 307) by Yang *et al*. used structural and dMRI data and reported 75% accuracy.^[145]^

Few recent studies have also focused on cross-disorder prediction of MDD and BP, as well as MDD and SZ. For instance, using multivariate patterns form gray matter differences and LOO-CV, Redlich and colleagues differentiated MDD from BP patients with 79.3% accuracy, and further validated the findings using an independent dataset and test–retest validation with 65.5% accuracy.^[157]^ He *et al*. used multimodal fusion analysis combined functional network and gray matter density and achieved high accuracy (90% accuracy) in discriminating across MDD, BP and HC groups.^[158]^ Burger *et al*. used emotion stimulation task and neural activation of the amygdala and the anterior cingulate gyrus to distinguish BP from MDD, the multivariate pattern classification analysis yielded significant classification rates of up to 72% based on ACG activation elicited by fearful faces.^[159]^ These lower accuracy in multi-label classification than the binary model would be mainly caused by the similar brain aberrances among subtypes of MPDs and heterogeneity in mental illness.

At present, however, there are two important limitations in the existing literature that limit the translational applicability of the findings in real world clinical practice. First, given the well-established effects of illness chronicity and antipsychotic medication on brain, it is unclear to what extent classification was based on ML features associated with these factors rather than the onset of the illness.^[160]^ Second, the clinical utility of any ML-based diagnostic tool was limited, which was focused on identifying disease-specific mechanisms and classification models.^[161]^ The generalization of the current ML findings were restricted. The third one is the reliability and stability of ML models which should be trained by dataset with larger sample size and be validated by independent study. Thus, we suggest future ML studies in mental illness should to acquire sufficient sample sizes training data and validation data, and meanwhile fully consider the importance of clinical variables among cross-sectional designs.

**Deep leaning methods:** Because the etiology of mental illness is highly diverse, brain features and ML models are highly complex systems involving multiple levels of temporal and spatial granularity and millions of nonlinear feedback loops. There have been high hopes recently that artificial intelligence (AI) algorithms, in particular from the field of DL can meet these challenges. DL algorithms excel in processing highly complex data within which data features may interact at multiple levels and in highly nonlinear ways.^[162]^ Deep neural networks (DNNs) can infer suitable high-level representations without much domain-specific knowledge and prior feature construction.^[163]^ This is due to their multi-layered design, where highly complex and intricate nonlinear relations among input features could be extracted and represented by layers further up in the processing hierarchy.

Most studies employing DL in psychiatry have focused on diagnostics.^[164]^ DL method was used in identifying brain abnormalities and diagnosis in both sMRI and rsfMRI studies.^[165,166]^ In a multicenter study, Zeng *et al*. used deep discriminant autoencoder network and functional connectivity features from 7 data centers to discriminate schizophrenic individuals from healthy controls, they had 81% accuracy by cross data site validation.^[167]^ In another dynamic FC study, Yan *et al*. used RNN method to discriminate schizophrenic individuals from healthy controls in 7 data centers, and they also got 80% accuracy and had higher accurate performances the SVM and Random Forest methods.^[168]^ In schizophrenia outcome prediction study, they found better overall performances (70% accuracy for the binary) than traditional six ML methods. There is lack of brain structural features for DL study of schizophrenia classification, which suggested the more informatic features.

#### Unsupervised learning methods for subtyping major psychiatric disorders

**Cluster analysis for subtyping:** Insightfully, the recent two works of Winter *et al*. and Chekroud *et al*. tacitly tested the accuracy of supervised learning method in predicting clinical diagnoses or treatment effectiveness regarding MPDs.^[169,170]^ Regretfully, despite the endeavors of both methodologies and data resources that have been made, neither of them have achieved satisfying model performance. These thought-provoking findings led us to rethink the essential dilemma of MPDs. Despite the widespread use of supervised ML approach in MPDs classification, for the strongly heterogeneous MPDs population, seeking a single neurophysiological signature would be incapable of guiding its clinical diagnosis and treatment.^[171]^ The heterogeneity of MPDs stems from its symptom-based diagnostic criteria.^[117]^ Taking MDD as an example, patients who present with one identical clinical manifestation such as anhedonia could be driven by different underlying etiologies ranging from altered dorsomedial prefrontal cortical connectivity to dysfunctional basal ganglia.^[172,173]^ Besides, the diagnosis of MDD requires a patient to report at least 5 of 9 symptoms; this leads to some 1500 DSM-IV symptom combinations, further increasing the heterogeneity of this confounding group.^[174]^ To sum up, the clinical practice of MPDs are plagued by the complicated syndromic diagnostic classification; consequently, identifying disease subtypes *via* an objective pattern would yield a new insight withal.

Neuroimaging could potentially reflect the underlying neural mechanisms of MPDs and serves as an ideal intermediate phenotype to define the heterogeneous MPDs patients into homogeneous subtypes. As an unsupervised learning approach, cluster analysis is increasingly applied in neuroimaging studies for MPDs subtyping. However, unlike the supervised ML, there is lack of class labels in cluster analysis. Slight changes in samples, neuroimaging features or clustering algorithms could significantly alter the clustering results, adding to great difficulties and challenges in cluster analysis. Most clustering studies concerning MPDs subtyping hence present with latent deficits to some extent, which broadly leads to a weak reliability in current findings.

**Cluster reproducibility:** One considerably important concept that most studies either neglected or incorrectly used in clustering for MPDs subtyping would be the cluster reproducibility. Here we delimitate it into the three following aspects, namely the reproducibility of clustering algorithms, results and features. Firstly, considering that cluster analysis focuses on the properties of clustering problems rather than algorithms, discussing the reproducibility of the clustering algorithm would be meaningless for MPDs subtyping.^[175]^ Giving Price *et al*.’s two studies as examples, the authors repeatedly delivered the community detection algorithm on connectivity maps generated through Subgroup-Group Iterative Multiple Model Estimation (S-GIMME) to identify MDD neuroimaging subtypes based on fMRI data; both typical and atypical subtypes were found in each study. Yet the subtypes between studies showed no significant overlap.^[176,177]^ The failure of reproduction implied their subtyping to be unreliable.

Furthermore, several studies began to take notice of the reproducibility of clustering results; Chand *et al*. used the adjusted Rand Index (ARI) for reproducibility evaluation, suggesting that higher ARI represented more reproducible clustering results.^[178]^ However, given the nature of lacking class labels in cluster analysis, one global clustering quality score does not actually exist for clustering problems. In fact, the very concept of reproducible clustering results would be ambiguous; instead, for MPDs subtyping, the clustering reproducibility rests with a reproducible neuroimaging feature capable of dividing MPDs into stable neuroimaging subtypes.

However, the uniqueness of neuroimaging studies as well as the widespread misuses of supervised learning techniques in unsupervised studies lead to a limited reproducibility of neuroimaging features for MPDs subtyping. To begin with, the small sample sizes of neuroimaging studies significantly impede subtyping exploration and validation. For instance, Dwyer *et al*. identified neuroanatomical subtypes of SCZ using sMRI data. Yet their discoveries were limited to two subtypes due to the inadequate sample size for further downstream evaluation.^[179]^ Queries were consequently raised on their SCZ subtypes, and as a result, no reproducible neuroanatomical features were identified during external validation.

Secondly, the neuroimaging data are generally with high dimensionality; for example, due to differences in acquisition resolutions and regions of interests, the number of nodes in brain networks could range roughly from 3400 to 140,000, suggesting a neuroimaging data reaching up to 140,000-dimensional.^[180]^ The high dimensionality will lead to a common obstacle in ML named “curse of dimensionality”, in which circumstance a natural strategy is dimensionality reduction.^[181]^ In the famous work of Drysdale *et al*., the canonical correlation analysis (CCA) was used to correlate the FC with clinical symptoms and the high-dimensional FC data were reduced into low-dimensional space.^[182]^ Nevertheless, the CCA method was potentially limited by overfitting; resulted from the inappropriate dimension reduction method, and the identified anhedonia-and anxiety-related connectivity features revealed poor reproducibility.^[183]^ Notably, one recent progress has been made regarding their work by optimizing CCA into a regularized CCA approach, while its robustness requires further validation in more independent centers.^[184]^

Last but not least, the misconceptions of disease subtyping, clustering and classification as well as the inappropriate application of machine-learning techniques widely exist in psychiatry. Recently, Winter *et al*. proposed a pipeline for MPDs subtyping based on cluster analysis, including generalization, testing of statistical significance, avoidance of overfitting, and CV.^[185]^ However, clustering is fundamentally distinct from classification due to the lack of class labels and the unknown data distribution; hence, their recommended methods developed within supervised learning cannot apply.^[186]^ The broad misconception and misuse of classification techniques in clustering lead to basic defects in current studies; as a result, most identified neuroimaging signatures of MPDs subtypes fail to reproduce.

**Cluster validity: Why subtyping?** Seeking a reproducible neuroimaging feature does not necessarily represent the clustering to be valid. Before further discussion, it would be of great significance to clarify the two different concepts: clustering and clustering for subtyping. Clustering is an unsupervised learning approach and there is no universal answer to clustering; clustering could be measured valid as long as it satisfies the need of study. By delivering cluster analysis, the psychiatrists aim at disease subtyping. Consequently, to achieve valid clustering, we should reemphasize the core question generally overlooked by psychiatrists: what’s the ultimate goal of subtyping? In view of the rapid development of modern medicine represented by oncology, the fundamental problem of psychiatry could be attributed to the lack of biomarker for developing accurate diagnosis and treatment.^[187]^ As such, by MPDs subtyping, we seek to identify an etiology-based objective neurobiomarker capable of unveiling disease neural mechanisms and guiding targeted interventions.

Considering the shared neuroimaging signatures across MPDs and the distinctions in different study cohorts, we developed a novel method for identifying MPDs transdiagnostic neurophysiological subtypes *via* rs-fMRI data; specifically, the deep stacked AutoEncoder was used to extract low-dimensional ALFF features followed by an ensemble clustering method for subtype identification.^[105]^ We identified the archetypal and atypical MPDs neuroimaging subtypes which revealed converse frontal-posterior functional imbalance patterns, and validated the subtypes using cortical thickness, white matter integrity, polygenic risk scores, and tissue profile for risk gene expression. Based on the frontal-posterior functional imbalance pattern, we further identified novel repetitive transcranial magnetic stimulation (rTMS) targets for the archetypal and atypical subtypes and conducted a clinical trial in two other independent centers.^[188]^ We hypothesized that if one brain region could effectively differentiate the MPD subtype from HC, then it should be the core dysfunctional brain region and could hence be potentially identified as subtype-specific rTMS target. Intriguingly, the identified rTMS targets were separately located in frontal and posterior brain regions, with the dorsomedial PFC being demonstrated as subtype-specific rTMS target for archetypal MPD, while the occipital cortex for atypical MPD.

We next performed the subtyping and precise rTMS strategy on hospitalized, depressed youths; post precise rTMS, both neuroimaging deficits and clinical symptoms were significantly improved, suggesting the frontal-posterior functional imbalance as an objective neurobiomarker to guide clinical practice. Furthermore, employing the neuroimaging as a cross-species intermediate phenotype, we separately established the methylazoxymethanol acetate (MAM) model as well as CUMS model which showed frontal-posterior functional imbalance patterns similar to the archetypal and atypical MPDs, and performed subtype-specific rTMS for mechanism exploration.^[189, 190, 191]^ Post intervention, the functional deficits in both subtypes were normalized, followed by structural and frontal synapse-associated proteomic alterations. We next translated our prior findings into a double-blinded, randomized controlled design (clinicaltrials.gov registration number: NCT05465928). Up to now, the clinical trial has been completed and the results are being reviewed. Taken together, we made an innovative attempt by identifying a transdiagnostic biomarker for disease subtyping and further translating it into precise rTMS to guide clinical practice. We expect experts in computer science to further make up for the methodological deficiencies during application of ML; by means of technical advances, we wish to build a neuroimaging-based framework beyond symptoms and eventually actualize precision medicine in Psychiatry.

## Conclusions and future directions: Chasing precision major psychiatric disorders

Remarkable evolutions have been made in psychiatric nosology by establishing a diagnostic classification framework to improve the diagnosis of psychiatric disorders. However, conventional diagnostic criteria of MPDs could be arbitrary due to the lack of accurate measures for drawing a clear diagnostic boundary and the limited etiologic understandings of the subjective symptoms, which further leads to a poor treatment outcome and significant disease burden. Fortunately, neuroimaging acts as an intermediate phenotype in the MPDs multi-level causal pathway, and advances in MRI techniques pave a new way for refining MPDs in a pathological approach. Accumulating MRI studies implicate both structural and functional neuroimaging commonalities and overlaps across MPDs with a decreasing severity trend of SCZ, BD and MDD. Specifically, multi-modal neuroimaging studies including sMRI, DTI and fMRI similarly revealed opposite changing patterns between prefrontal-subcortical brain regions and primary sensory and motor cortices, indicating the imbalanced alterations across a higher order-primary gradient to be the shared core neuroimaging deficits among MPDs. On the other hand, strong heterogeneity within MPDs and inconsistent results between different studies suggest the existence of MPDs neuroimaging subtypes with distinct underlying neural mechanisms. Developments of ML approach promote more individualized analyses of the complex MPDs neuroimaging data and its clinical translation. Using cluster analysis, we identified and validated the frontal-posterior functional imbalance as a transdiagnostic objective neurobiomarker to guide precise subtyping, treatment and evaluation of the MPDs in a closed-loop. Larger MPDs populations with more diverse racial components, advanced ML methodologies, randomized controlled designs and deepened mechanism explorations are needed in future studies to actualize a neuroimaging-based precision medicine framework in psychiatry.
